# Concentrated Growth Factors Promote hBMSCs Osteogenic Differentiation in a Co-Culture System With HUVECs

**DOI:** 10.3389/fbioe.2022.837295

**Published:** 2022-03-21

**Authors:** Yunyang Liao, Youran Fang, Hanghang Zhu, Yue Huang, Gengsen Zou, Bowen Dai, Macro Aoqi Rausch, Bin Shi

**Affiliations:** ^1^ Department of Oral and Maxillofacial Surgery, The First Affiliated Hospital, Fujian Medical University, Fuzhou, China; ^2^ Laboratory of Facial Plastic and Reconstruction, Fujian Medical University, Fuzhou, China; ^3^ Fujian Key Laboratory of Oral Diseases, School and Hospital of Stomatology, Fujian Medical University, Fuzhou, China; ^4^ Division of Orthodontics, University Clinic of Dentistry, Medical University of Vienna, Vienna, Austria

**Keywords:** Concentrated Growth Factors (CGF), cell co-culture, hBMSCs, osteogenic differentiation, connexin 43 (cx43)

## Abstract

Osteogenesis is a complex physiologic process that occurs during bone regeneration. This process requires several growth factors that act on bone marrow-derived mesenchymal stem cells (BMSCs). Concentrated growth factor (CGF) is a new-generation platelet-rich derivative that is an appealing autologous material for application in tissue repair and bone regenerative medicine because it contains a variety of fibrin and growth factors. In this study, the effects of CGF on the proliferation and osteogenic differentiation of hBMSCs and human umbilical vein endothelial cells (HUVECs) were explored with *in vitro* cell co-culture experiments. HBMSCs and HUVECs were directly co-cultured at the ratio of 1:2 under different concentrations (0, 2, 5, 10, 20%) of CGF for 7 days. Alkaline phosphatase (ALP) staining and quantitative reverse transcription polymerase chain reaction were used to detect the effects of CGF on the expression of osteogenic genes (ALP, osteocalcin [OCN], type I collagen [COL-1], Runt-related transcription factor 2 [RUNX2]) and connexin 43 (CX43). RNA sequencing was used to explore potential regulated genes and signaling pathways that affect the osteogenesis of co-cultured hBMSCs exposed to CGF. The results showed higher expressions of ALP, COL-1, RUNX2, OCN, and CX43 in the direct co-culture group containing 10% CGF compared to the direct co-culture group without CGF and the indirect co-culture group. In summary, 10% CGF can significantly promote osteogenesis in hBMSCs directly co-cultured with HUVECs. Intercellular communication between the direct co-culture of hBMSCs and HUVECs through CX43 may be one of the main regulatory mechanisms.

## Introduction

Repairing bone defects is still challenging for clinicians. In bone tissue engineering, bone marrow-derived mesenchymal stem cells (BMSCs) with regenerative potential are considered an ideal cell source for bone regeneration, as they show promise in the treatment of bone loss and osteogenesis imperfecta (Bajada et al., 2008; Zhang et al., 2018). An important strategy for effective bone regeneration is to introduce BMSCs expanded *in vitro* to the affected area and ensure their differentiation into functional bone matrix-forming cells (Hofmann et al., 2008). MSCs possess an excellent ability of multi-differentiation, properties of immunosuppressive as well as a range of biologically active macromolecules that the establishment of regenerative microenvironment needs (Turnovcova et al., 2009).However, the limitation is that the proportion of BMSCs in adults is fairly low, while a large number of BMSCs is required for the treatment (Sotiropoulou et al., 2006). Studies have shown that the development of BMSCs can be determined by the following factors: exogenous soluble growth factors, cytokines, hormones and chemicals, as well as external mechanical adjustment (Alsberg et al., 2006). Concentrated growth factor (CGF) is the third-generation platelet-rich derivative after platelet-rich plasma and platelet-rich fibrin; it contains fibrin and a variety of growth factors such as platelet-derived growth factor, vascular endothelial growth factor, and transforming growth factor-β1. These components give CGF excellent tissue repair and regeneration abilities (Yu and Wang, 2014; Zhang and Ai, 2019). Another way to determine the development of BMSCs is to rely on co-culture with other mature cell populations (Ball et al., 2004). Endothelial cells are used to promote angiogenesis; when they are in direct contact with BMSCs they can successfully induce osteogenic differentiation *in vitro* by expressing various factors (Kaigler et al., 2005; Chen et al., 2018). Studying co-cultured human BMSCs (hBMSCs) and human umbilical vein endothelial cells (HUVECs) is therefore useful for evaluating the correlation between osteogenesis and angiogenesis in the human body, as well as great significance for exploring the application of hBMSCs for bone regeneration. This study used the direct and indirect co-culture system of hBMSCs and HUVECs to study the effect of CGF on hBMSC osteogenic differentiation. Analyzing the differences of osteogenic expression and the mechanics under both direct and indirect co-cultures is of great practical value to promote clinical bone regeneration and bone healing.

## Materials and Methods

### CGF preparation

This study was approved by the Ethics Committee of the First Affiliated Hospital of Fujian Medical University, Fuzhou, China (27 June 2020; MRCTA, ECFAH of FMU [2020]300), and informed consent was obtained from the donors. Venous blood was obtained from healthy, nonsmoking volunteers aged from 25 to 35 years old. Blood samples were collected into the CGF exclusive vacutainers with red caps and centrifuged according to the preset protocol of the Medifuge centrifuge (Thermo Fisher Scientific, Waltham, MA, United States). After 30 s, the machine sped up to 2,700 rpm for 2 min, 2,400 rpm for 4 min, 2,700 rpm for 4 min, and 3,000 rpm for 3 min. The speed was finally decreased to 0 rpm after 36 s, and the product was divided into three layers. At the top was platelet-poor plasma, fibrin and CGF were in the middle, and the bottom layer contained red blood cells and platelets.

When the upper platelet-poor plasma had been discarded, the CGF clot was collected from the tube with sterile tweezers. Next, the yellow gel and red part of the interface were carefully cut with sterile scissors and crushed in the cryotubes. They were then soaked in liquid nitrogen (5 min) and followed by 37°C warm water for 5 min each three times. The following stage was a 20-min centrifugation at 1,000 *g*, so the cryosupernatant (CGF extract) was collected. After filtration, the product was frozen at −80°C (Qiao et al., 2017). We prepared 2, 5, 10, and 20% concentrations of CGF from the 100% stock by dilution with ECM. 0% CGF is ECM with no CGF.

### Cell Culture

#### Cell Subculture

Both hBMSCs and HUVECs were purchased from Sciencell (Carlsbad, CA, United States). The cells were seeded at 5,000 cells/cm^2^ in culture bottles treated with bovine plasma fibronectin (Sciencell). ECM culture medium (Sciencell) containing 5% fetal bovine serum (Gibco, Grand Island, NY, United States), 1% penicillin mixture, and 1% endothelial cell growth factor (Sciencell) was used for HUVECs. HBMSCs were cultured in mesenchymal stem cell medium (Sciencell) containing 5% fetal bovine serum, 1% penicillin mixture, and 1% mesenchymal stem cell growth factor. The cells were placed in a constant temperature incubator with 5% CO_2_ at 37°C to subculture. The third to fifth passages of cells were used in the experiments.

#### Direct Cell Co-culture

The cells were seeded into six-well plates (hBMSCs: 3,500 cells/cm^2^, HUVECs: 7,000 cells/cm^2^). There were five groups according to the different concentrations of CGF (0, 2, 5, 10, and 20%). The plates were cultured under 5% CO_2_ at 37°C. After 12 h when cells had adhered to the wall, the original media were replaced with ECM media containing different CGF concentrations for further culture. The medium was changed every 3 days. All experiments were performed in triplicate.

#### Indirect Cell Co-culture

Using the numbers of cells as for direct co-culture, hBMSCs were seeded at the bottom of six-well plates. After the Transwell chambers were placed into cell culture holes, HUVECs were seeded in the chamber. The plates were then cultured under 5% CO_2_ at 37 °C. After 12 h when cells had adhered to the wall, the original media were replaced with ECM media containing 10% CGF. Both the direct and indirect co-culture control groups were replenished with ECM medium without CGF and further cultured in the constant temperature incubator. The medium was changed every 3 days. On day 7, the cells at the bottom of the six-well plates and in the Transwell chambers in the indirect co-culture groups were digested and collected. All experiments were performed in triplicate.

### Proliferation Assay

On days 1, 4, and 7 of direct cell co-culture with CGF, the medium was discarded, and 0.1% type IV collagenase (Gibco) was added. This was then digested in the incubator with 5% CO_2_ at 37°C for 30 min. After another rinse in phosphate-buffered saline (PBS), 0.25% trypsin was added and digested again at 37°C for 1–2 min to completely disperse the cells. Complete medium (3–4 times the volume of trypsin) was added to terminate digestion. After this, single cell suspensions were collected and centrifuged at 1,000 rpm for 5 min. The cryosupernatant was discarded, PBS was added to resuspend cells, and the total number of cells was counted. This was followed by another centrifugation and the addition of 100 μL PBS and 5 μL anti-human CD31 FITC (eBioscience, San Diego, CA, United States). After incubation on ice for 30 min in the dark, 500 μL PBS was added to resuspend to single cell suspension. Flow cytometry was used to count the proportion of different cells. CD31-positive cells were defined as HUVECs and CD31 negative cells as hBMSCs. The proportions of positive and negative cells were multiplied respectively by the total number of cells to quantify the numbers of HUVECs and hBMSCs in each group.

### Alkaline Phosphatase Staining and ALP Activity Assay

The culture media was discarded on day 7 of direct cell co-culture with CGF. The cells were washed three times in PBS, then fixed at room temperature with 4% paraformaldehyde for 30 min. The fixative was then discarded, and the cells were rinsed three more times in PBS. After addition of BCIP/NBT (5-bromo-4-chloro-3′-indolyphosphate p-toluidine salt/nitro-blue tetrazolium chloride) solution (Beyotime, Shanghai,China), the cells were incubated at room temperature in the dark for 30 min or longer.Distilled water was used to wash the cells twice to stop the reaction. ALP staining was observed and photographed under the inverted microscope.

Before the ALP activity measurement, the protein content in each cell lysate samples was quantified by a BCA kit (Beyotime).And then the ALP activity assays were performed with Alkaline phosphatase Assay Kit (Beyotime) according to the manufacturer’s instructions.

### Examination of Osteogenesis-Related Genes

Cells were collected by centrifugation on day 7 of cell co-culture with CGF, and TRIzol lysate was added to isolate total RNA. cDNA was synthesized according to the instructions of PrimeScript™ RT reagent Kit with gDNA Eraser Kit (Takara Bio Inc., Japan). The SYBR Premix Ex Taq™ kit (Takara Bio Inc.) was also used following the manufacturer’s protocol to amplify osteogenesis-related genes through quantitative reverse transcription polymerase chain reaction (qRT-PCR). The osteogenesis-related marker genes examined were Runt-related transcription factor (Runx2), type I collagen (Col-I), osteopontin (OPN), and osteocalcin (OCN). Glyceraldehyde-3-phosphate dehydrogenase (GAPDH) was used as the housekeeping gene to normalize RNA expression levels. The PCR primer sequences are displayed in Table 1.

**TABLE 1:** Primer sequences of qRCR’

Gene	Prime sequences
GAPDH	Foward: GGT​GTG​AAC​CAT​GAG​AAG​TAT​GA
	Reverse:GAGTCCTTCCACGATACCAAAG
COL-1	Foward: GAG​AGC​ATG​ACC​GAT​GGA​TTC
	Reverse: CTT​CTT​GAG​GTT​GCC​AGT​CTG
ALP	Foward: CTG​GAC​CTC​GTT​GAC​ACC​TG
	Reverse: TCC​GTC​ACG​TTG​TTC​CTG​TT
RUNX2	Foward: CAC​CAC​TCA​CTA​CCA​CAC​CTA
	Reverse: TGA​CGA​AGT​GCC​ATA​GTA​GAG​AT
OPG	Foward: ACG​CAC​TAA​AGC​ACT​CAA​AGA​C
	Reverse:ACTGATTGGACCTGGTTACCTATC
CX43	Foward: CCT​GTA​TGC​CAA​CAT​TTG​CTT​TC
	Reverse: GGT​GCT​TCC​TCC​TTT​CAT​CAG
OCN	Foward: ATT​GAC​GGG​TAC​CTC​TCC​CA
	Reverse: TCC​AGG​TCA​CAG​GTC​TCG​AA
PIK3CA	Foward: AGAGCCCCGAGCGTTT
	Reverse: TCG​TGG​AGG​CAT​TGT​TCT​GA
MAP2K1	Foward: GCG​TTA​CCC​GGG​TCC​AAA​AT
	Reverse: TCCAAGTTGGTCTCCGCA
ULK1	Foward: TCC​AAA​CAC​CTC​GGT​CCT​CT
	Reverse: AAC​TTG​AGG​AGA​TGG​CGT​GT

### Immunofluorescence Staining

The third passages of hBMSCs and HUVECs were collected. Sterile cover slides were placed in 12-well plates. At the densities of 3,500 cells/cm^2^ for hBMSCs and 7,000 cells/cm^2^ for HUVECs, the cells were mixed and seeded into the plate. The cells were cultured on the slide in a constant temperature incubator with 5% CO_2_ at 37 °C. The medium was changed every 3 days. On day 7, the cells were washed three times in PBS and then fixed in 4% paraformaldehyde at room temperature for 30 min. This was followed by three more rinses in precooled PBS before the cells were blocked with 10% goat serum. After incubation at 37 °C for 30 min, the plate was turned over to dispose of the excess serum. Next, 100 μL of primary antibody (CD31 rabbit anti-human protein and connexin 43 (CX43) mouse anti-human protein antibody (both from Invitrogen, Carlsbad, CA, United States) (at titers of 1:60 and 1:100 respectively) was added and incubated at 4 °C overnight. After three more rinses in PBS, 100 μL of secondary antibody (Alexa Fluor488 goat anti-rabbit, Alexa Fluor647 goat anti-mouse, at titers of 1:200), then they were incubated at room temperature for 1 h before three washes in PBS. DAPI (4′,6-diamidino-2-phenylindole) staining solution was added for incubation at room temperature in the dark for 10 min, followed by three rinses in PBS. Finally, the slides were coated with drops of sealant, and the covering glass was placed upside down on the sealant using a pair of tweezers. Slides were then observed and photographed under a fluorescence microscope (IX-71, Olympus, Japan).

### RNA Sequence and Bioinformatics Analysis

Total RNA was extracted from directly co-cultured hBMSCs in the 10% CGF and 0% CGF groups. Complementary DNA libraries were sequenced on an Illumina NovaSeq platform (Illumina, San Diego, CA, United States) to generate 150-bp paired-end reads, according to the manufacturer’s instructions. Differential expression analysis between the two conditions was performed using the DEGSeq R package (1.20.0). Differentially expressed genes (DEGS) were defined as those with adjusted P-values below 0.05 and log2 (fold change) > 1. Gene Ontology (GO) and Kyoto Encyclopedia of Genes and Genomes (KEGG) enrichment analyses of DEG sets were implemented in the GOseq R and KOBAS 3.0 package, respectively. GO terms with adjusted P-values below 0.05 were considered as significantly enriched by DEGs.

### Data Analysis

SPSS 22.0 (IBM Corp., Armonk, NY, United States) was used for analyzing and processing data, which are expressed as mean ± standard deviation. The normality and homogeneity of variance of each group were examined, then one-way analysis of variance was used to compare the data of each group at the same time point. Differences were considered significant at *p* < 0.05.

## Results

### Effects of CGF on the Proliferation of Directly Co-cultured hBMSCs and HUVECs

**FIGURE 1:**
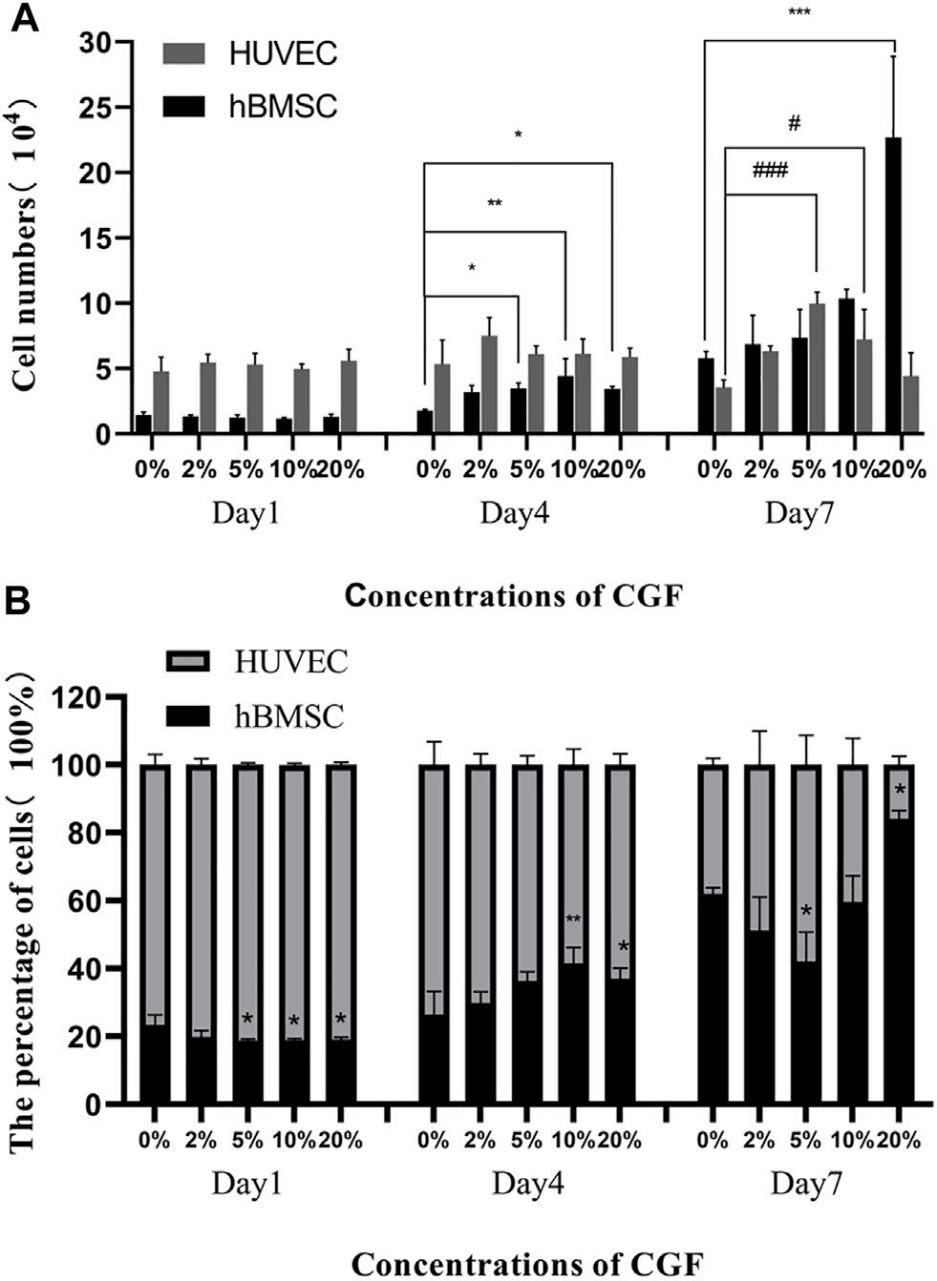
The number of hBMSC and HUVEC directly co-cultured at a ratio of 1:2 on the day 1, day 4 and day 7 under different concentrations of CGF (hBMSC: ^*^ *p* < 0.05, ^**^ *p* < 0.01, HUVEC: ^#^ *p* < 0.05, ^###^ *p* < 0.001, 0%: co-cultured control group). **(A)**: numbers of the 2 cells on the 1^st^, 4^th^, and 7^th^ day. **(B)**: percentages of the 2 cells on the 1^st^, 4^th^, and 7^th^ day.

hBMSC proliferation showed CGF concentration-dependent changes over time, but HUVEC proliferation was not obvious. On day 4, the numbers of hBMSCs in the 5, 10, and 20% CGF groups were significantly higher than that in the co-cultured control group (*p* < 0.05). When there was <10% CGF, the number of hBMSCs increased with higher CGF concentrations, but the proliferation level of hBMSCs in 20% CGF was lower than the level under 10% CGF. On day 7, only the number of hBMSCs in the 20% CGF group was significantly higher than that in the co-cultured control group (*p* < 0.05), and the number of hBMSCs increased with greater CGF concentrations. With regard to HUVECs, there was no evident change in the number on days 1 and 4. On day 7, the numbers of cells in the 5 and 10% CGF groups were notably higher than that in the co-cultured control group (*p* < 0.05). When the CGF concentration was <5%, the number of HUVECs rose with higher concentrations of CGF. (Figure 1A).

On day 1 of CGF treatment, the percentages of hBMSCs in the 5, 10, and 20% CGF groups were notably lower than in the co-cultured control group (*p* < 0.05). However, on day 4, the percentages of hBMSCs in the 10 and 20% CGF groups were significantly higher than that in the co-cultured control group (*p* < 0.05), and the percentage of hBMSCs in 10% CGF was the highest among all the groups. On day 7, the percentages of hBMSCs were lowest and highest in the 5 and 20% CGF groups, respectively. The differences were statistically significant compared with the control group (*p* < 0.05, Figure 1B).

### Effects of CGF on Osteogenic Differentiation of hBMSCs in Direct Co-culture

**FIGURE 2:**
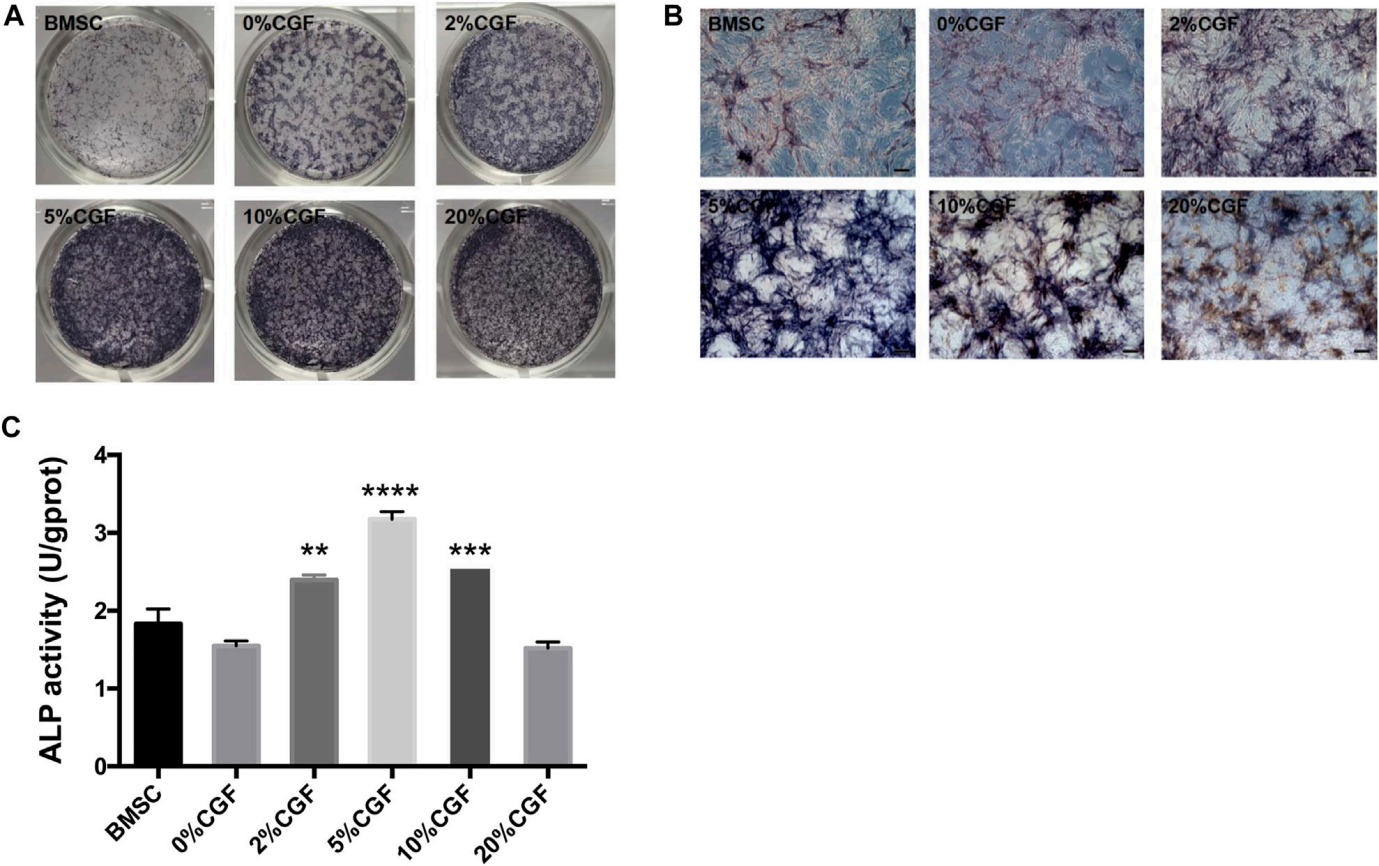
Alkaline phosphatase (ALP) staining and its quantitative analysis.ALP staining and activity was detected in single cultured hBMSCs group (BMSC) and five directly co-cultured group with different concentrations of CGF (0, 2, 5, 10, and 20%), for 7 days. **(A)**: General observation. **(B)**: ALP staining under fluorescence microscope (ruler: 200 μm). **(C)**: ALP quantitative analysis (*p < 0.05, **p < 0.01, ***p < 0.001, ****p < 0.0001 versus 0%CGF).

To examine the early influence of different concentrations of CGF on the osteogenic differentiation ability of directly cultured hBMSCs, we performed ALP staining and the ALP activity for qualitative and quantitative analyses. Staining was performed after 7 days of CGF treatment. The staining results showed increased staining of each group treated with CGF compared to untreated hBMSCs. Notably, when the CGF concentration was <10%, the color deepened with increased CGF concentration. However, a 20% CGF, the staining became lighter (Figures 2A,B). And its quantitative analysis also indicated that the presence of CGF caused the increase in the ALP activity when the CGF concentration was <10%(Figure 2C).

### Effects of CGF on Osteogenic-Related Gene Expression of hBMSCs in Direct Co-culture

**FIGURE 3:**
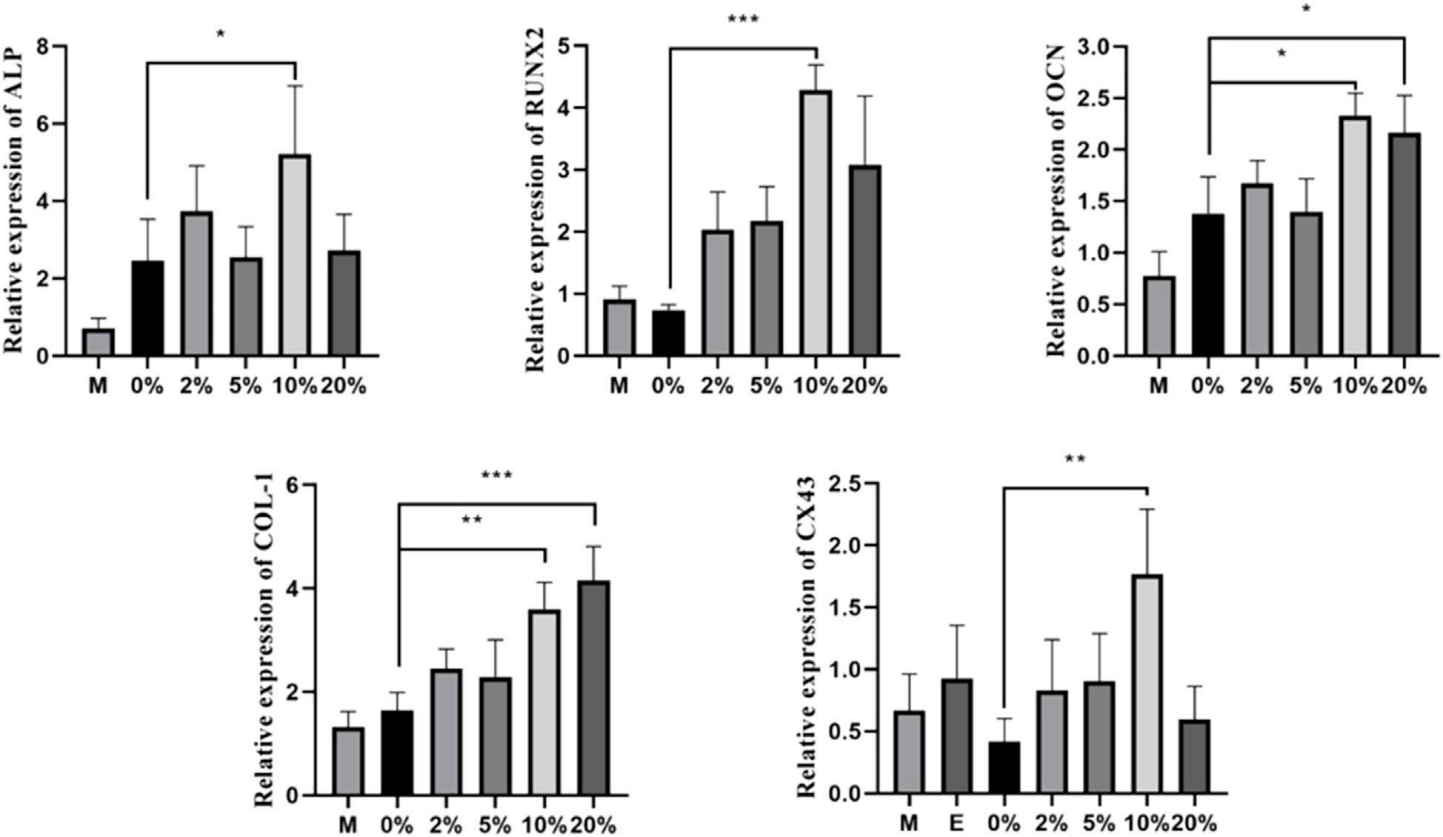
Under different CGF concentrations, the relative expressions of osteogenic-related genes of hBMSCs in direct co-culture (*p < 0.05, **p < 0.01, ***p < 0.001, ****p < 0.0001). (M: the group of single-cultured hBMSCs without CGF; E: the group of single-cultured HUVECs without CGF).

To compare relative gene expression levels under different CGF concentrations, we measured the osteogenic-related genes ALP, OCN, COL-1, Runx2, and CX43 using qRT-PCR after 7 days of 2, 5, 10, and 20% CGF treatment. In the 10% CGF group, the expression levels of ALP, Col-1, Runx2, OCN, and CX43 were all higher than those in the co-cultured control group (*p* < 0.05). Furthermore, the promotive effect of 10% CGF on the expressions of ALP, RUNX2, OCN, and CX43 was higher than all other groups, with only the effect on COL-1 gene expression being second to 20% CGF. Overall, 10% CGF most effectively promoted osteogenic differentiation of direct co-cultured hBMSCs and cross-linking between hBMSCs and HUVECs. Moreover, the expression levels of ALP, COL-1, and OCN in co-culture groups were higher than those with only hBMSCs. In comparison with the co-cultured control group, COL-1 and OCN also exhibited evident increases under 20% CGF (*p* < 0.05, Figure 3).

### CX43 Expression in hBMSCs and HUVECs in Direct Co-culture

**FIGURE 4:**
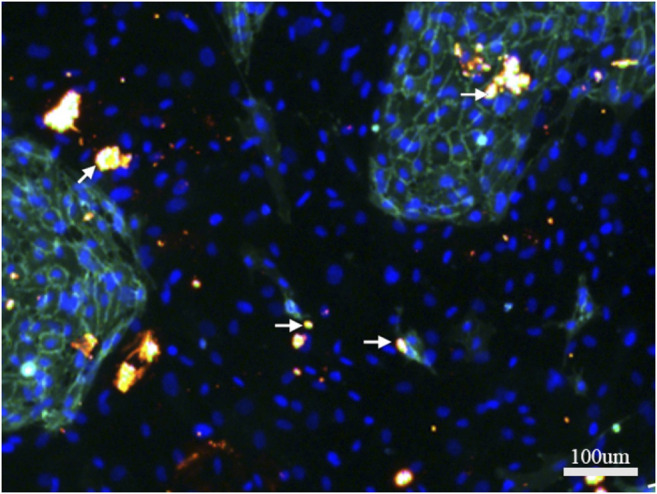
Immunofluorescence staining of CX43 between hBMSCs and HUVECs in direct co-culture. CX43 (orange) to label the linker protein, CD31 (green) to label HUVECS, DAPI (blue) to show nuclei.White arrows indicate the CX43 expression between hBMSCs and HUVECs.

CX43 was immunofluorescently stained on day 7 of directly co-culturing hBMSCs and HUVECs. Bright, dotted staining was observed as orange dots connecting hBMSCs and HUVECs (Figure 4). The results suggested the formation of gap junctions between hBMSCs and HUVECs, as well as between hBMSCs and HUVECs.

### Effects of 10% CGF on hBMSCs and HUVEC Osteogenic-Related Gene Expression in Direct and Indirect Co-cultures

**FIGURE 5:**
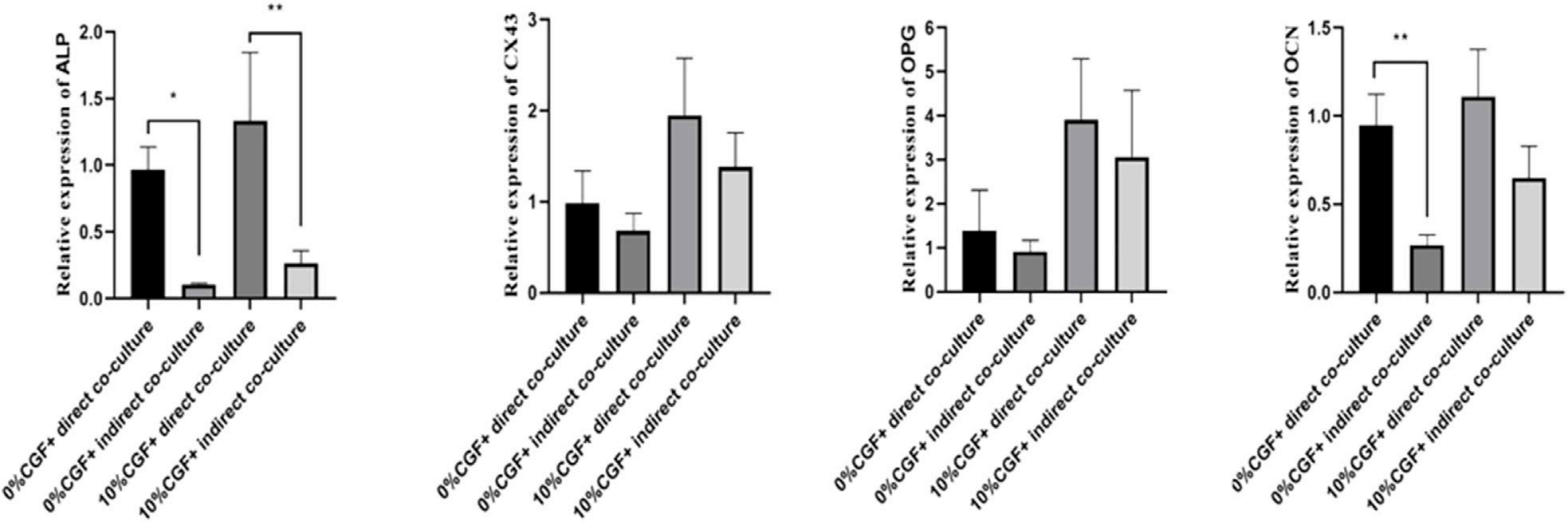
Under 10% CGF, the relative expression of hBMSCs osteogenic-related genes in direct and indirect co-culture with HUVECs (*p < 0.05, **p < 0.01, ***p < 0.001, ****p < 0.0001).

After 7 days of treatment, ALP expression in both direct co-culture groups and the control group was higher than in the indirect culture groups (*p* < 0.05, Figure 5). Although the differences were not significant, the expressions of OPG, OCN, and CX43 showed the same trend. The increases in the direct co-culture and control groups were more evident than in the indirect co-culture and control groups. This indicated that direct co-culture groups were more effective in promoting the osteogenic differentiation of co-cultured cells than indirect co-culture groups, regardless of the presence of CGF. These results suggested that cell communication between hBMSCs and HUVECs played an important role in promoting the osteogenic differentiation of co-cultured cells. In addition, 10% CGF enhanced the expressions of ALP, OPG, OCN, and CX43 in both the direct and indirect co-culture groups compared to the control groups without CGF. Furthermore, the expressions of ALP, OPG, OCN, and CX43 in direct co-culture groups with 10% CGF were the highest, suggesting that CGF could facilitate the osteogenic differentiation of co-cultured cells. This could also be augmented by increased intercellular communication as a regulatory mechanism.

### RNA-Seq and Bioinformatics Analysis Results

**FIGURE 6:**
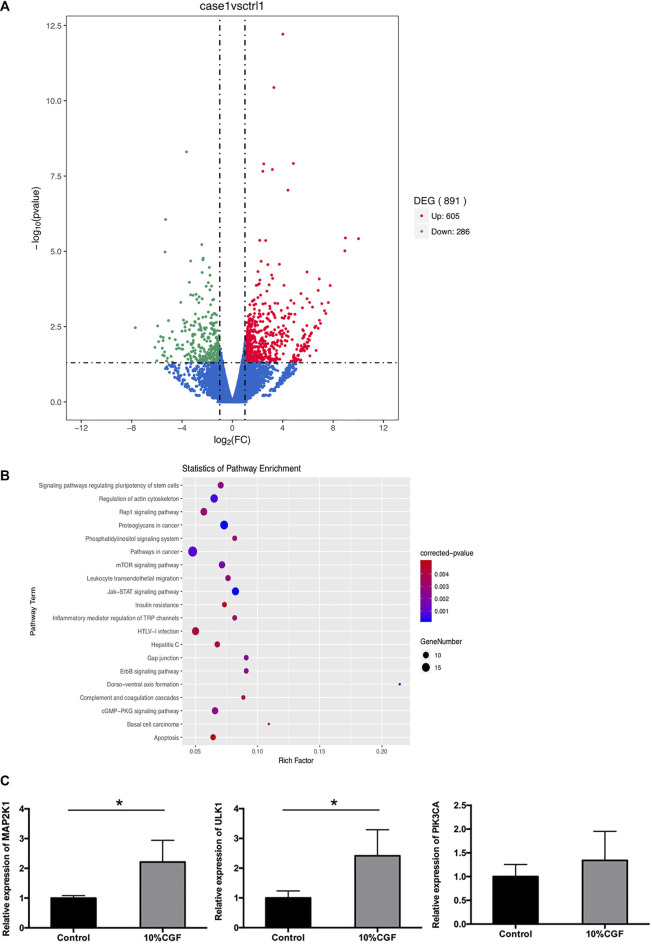
The results of RNA-seq, **(A)**: Volcano plot of differentially expressed genes (DEGs) between 10%CGF and control group in directly co-cultured hBMSCs, **(B)**: Representative up-regulated KEGG pathway categories, **(C)**: mRNA expression level of MAP2K1, ULK1, PIK3CA (*p < 0.05).

To explore the mechanism of pro-osteogenic differentiation of direct co-culture hBMSCs by CGF, we performed transcriptome analysis with high-throughput RNA-seq. The results showed that 605 DEGs were upregulated while 286 DEGs were downregulated in the 10% CGF group compared to 0% CGF group (Figure 6A). KEGG pathway analysis indicated that the phosphatidylinositol-3-kinase (PI3K)/AKT/mammalian target of rapamycin (mTOR) signaling pathway may play an important role in the effects of CGF on the osteogenesis of co-culture hBMSCs (Figure 6B). The upregulated genes associated with mTOR signaling were validated by qRT-PCR. Compared to the control groups without CGF, incubation with 10% CGF enhanced the expressions of MAP2K1, ULK1, and PIK3CA in the direct co-culture group (Figure 6C).

## Discussion

Bone repair and bone defect reconstruction are of crucial importance in the field of odontostomatology. There are now studies showing that CGF promotes bone regeneration combined with BMSCs *in vitro* and vivo (Chen et al., 2018; Rochira et al., 2020). In addition, bone repair and reconstruction are biological processes regulated by many cellular interactions and numerous signaling pathways (Kim et al., 2021). In an indirect co-culture system, the biological behavior of cells is only regulated by paracrine signaling whereas in direct co-culture system, cells are influenced by both paracrine and intercellular signaling between cells, which better reflects the actual situation compared to traditional single cell culture (Villars et al., 2000; Hu et al., 2020). It is an ideal experimental model to evaluate interactions between different cells. The literature describes increased osteogenic and angiogenic potentials of HVUECs directly co-cultured with mesenchymal stem cells (MSCs, at the ratio of 1:1) (Nguyen et al., 2020). In this study, different concentrations of CGF were applied to the direct and indirect co-culture of hBMSCs and HUVECs to elucidate the effect of CGF on the osteogenic differentiation of co-cultured hBMSCs. Because of the competitive growth among different cells in the co-culture system, the inoculation ratio is important. Our preliminary study showed that when the hBMSC:HUVEC ratio was 1:2, the numbers of the 2 cells were similar, which helped avoid the influence of too many cells of one kind on the accuracy of cell interaction evaluation.

Previous study have shown that the promotive effect ≤10% CGF on proliferation, migration and differentiation of HUVECs is positively correlated with CGF concentration (Bajaj et al., 2013). CGF concentrations between 1 and 10% promote the proliferation, osteogenic maturation, and mineralization of hTERTE6/E7 hBMSCs in a dose-dependent manner, but higher concentrations can be inhibitory (Hong et al., 2019). However, we found that when hBMSCs and HUVECs were directly co-cultured at a ratio of 1:2 with different concentrations of CGF, hBMSC proliferation in the CGF groups was enhanced to different extents at the early stage, whereas the number of HUVECs did not show notable changes. Over time, HUVEC proliferation was positively correlated with the CGF concentration at <5% CGF, but it was inhibited when the concentration was >5%. However, hBMSC proliferation was CGF concentration-dependent; it rose with higher CGF concentrations with no detectable signs of inhibition. We believe that the difference may be due to interactions between the two different cell types in direct co-culture, which changed the dependence of proliferation on CGF concentration in single-cell culture. Regardless of the CGF concentration, the proportion of hBMSCs increased with time, which may also be related to the promotive effect of HUVECs on hBMSC proliferation.

In addition to promoting angiogenesis, low-concentration CGF can also promote the proliferation and osteogenic differentiation of BMSCs in a dose-dependent manner. However, when the concentration of CGF exceeds 10%, the proliferation and differentiation of periosteal cells and mesenchymal stem cells (MSCs) is inhibited ^[2O]^. Nevertheless, another group reported that CGF could promote MC3T3-E1 osteoblast proliferation and differentiation, and 20% CGF was more effective than 10% CGF, as well as enhancing osteogenesis-related gene expression to a greater degree (Bubnov et al., 2013). In this case, the optimal concentration of CGF for promoting osteogenic or vascular differentiation varied greatly according to the type of cells. In our experiment, ALP staining was used to observe the effect of CGF with different concentrations on hBMSC bone formation ability after 7 days. ALP staining of the experimental groups with different CGF concentrations were all more intense than that of hBMSCs without CGF. Moreover, general and microscope observations clearly showed that when the CGF concentration was <10%, the color deepened in a CGF concentration-dependent manner. However, when the concentration of CGF reached 20%, the stain range decreased and became lighter. In addition, cell morphology revealed significant differences between single- and co-cultured hBMSCs. Single-cultured hBMSCs were randomly distributed on the cell culture surface. On day 7, they became fibrous and were arranged in parallel. Conversely, the cells in co-culture were distinctly rearranged in clusters and formed a tubular network. As the cell density increased, this structure became less apparent. These differences in morphology and staining were also obvious in the ALP staining results. Compared with hBMSC single-culture groups, CGF experimental groups exhibited increased ALP expression, and group with 5 and 10% CGF had the highest expression. This is consistent with the ALP staining results. Runx2 is an early transcription factor that has been proven to be crucial for osteoblast genesis (Bruderer et al., 2014; Querques et al., 2019). OCN is a non-collagenous calcium-binding osseous protein and a marker of late osteogenic differentiation (Ilmer et al., 2009). The expression levels of these two genes were dramatically raised under the effect of 10% CGF, which further confirmed the promotive function of 10% CGF in the differentiation of co-cultured hBMSCs along the osteoblastic lineage. COL-1 is an extracellular matrix protein that is a major organic component of bone tissue. It independently induces the expressions of OPN and ALP through FAK and ERK signaling (Viale-Bouroncle et al., 2014). The expression of COL-1 also evidently increased with 10% CGF. Moreover, the relative expressions of ALP, OCN, and COL-1 in co-culture were all higher than those in hBMSC single culture. Considering what has been discussed, hBMSCs co-cultured with HUVECs have stronger bone-forming ability. This ability even improves under the effect of 10% CGF.

In this study, the relative expressions of osteogenic genes in direct co-culture system were higher than that in the hBMSC single-culture system, this difference may be due to the behavior of cells being influenced by not only paracrine signaling but also direct communication between cells. CX43 is the most abundant connexin in the osteoblastic lineage and plays a key role in bone formation (Watkins et al., 2011). Many studies have shown that direct co-culture of osteoblasts and HUVECs enables the 2 cell types to form gap junctions through CX43. Only direct contact between cells through such gap junctions can promote osteoblastic differentiation (Villars et al., 2000; Guillotin et al., 2008; Herzog et al., 2014). Compared with other experimental groups with different CGF concentrations, we found that 10% CGF promoted the expression of bone-related genes and significantly enhanced the expression of CX43. This result suggested that the promotive effect of 10% CGF on hBMSC differentiation may be correlated with the direct contact of cells through gap junctions between the same or two different cell types. The formation of CX43 gap junctions between hBMSCs and HUVECs and between their heterotypic cells was also confirmed by immunofluorescence staining. Whether functional coupling between hBMSCs and HUVECs plays a key role in 10% CGF promoting hBMSC osteogenic differentiation requires further analysis of the differences of osteogenic gene expression in direct and indirect co-cultures. In this case, 0.4-μm Transwell chambers were used to indirectly co-culture the two kinds of cells. The wells prohibited cells to from contacting each other, so only paracrine signaling was exchanged between hBMSCs and HUVECs.

With 10% CGF, the expressions of the osteogenic genes ALP, OPG, and OCN were significantly higher in direct co-culture groups compared to indirect co-culture groups. In the 3D-sphere co-culture system of hBMSCs and HUVECs, the latter can both promote osteogenic differentiation and regulate hBMSC activity by activating endogenous WNT signaling and bone morphogenetic protein signaling (Saleh et al., 2011). Compared with indirect co-culture, the effects of co-culture with HUVECs on MSC proliferation and differentiation are more obvious than in direct co-culture (Böhrnsen and Schliephake, 2016). This effect at least partly depends on the formation of heterotypic cell contact between HUVECs and MSCs, and HUVECs co-culture reduces low serum-induced MSC apoptosis by increasing the phosphorylation and subsequent inactivation of the pro-apoptotic protein BAD (Steiner et al., 2012). Compared with indirect co-culture, the CX43 expression of cells in direct co-culture with 10% CGF was more obvious. Our immunofluorescence experiments confirmed the formation of CX43 junctions between hBMSCs and HUVECs. Two intercellular signaling channels were established between cells, which shifted the signal related to hBMSC bone formation. This altered the biological behavior of hBMSCs to promote their differentiation into osteoblasts. Collectively, our results indicate that the communication between hBMSCs and HUVECs via gap junctions likely plays an important regulatory role in promoting hBMSCs oteogenic differentiation in a co-culture system with HUVECs.

Our above-obtained results showed that CGF promotes hBMSCs osteogenic differentiation in a co-culture system, but the underlying mechanism is not clear. Various signaling pathways have been found to regulate the osteogenic differentiation of hBMSCs, including Notch signaling, Wnt/β-catenin, MAPK singaling,TGFβ, BMP-Smad singaling, Jak-STAT signaling, and Hedgehog signaling(AlMuraikhi et al., 2021; Pengjam et al., 2016 .). Therefore, we performed RNA-Seq and KEGG pathway analysis to further study the underlying mechanism. The results revealed that differentially expressed genes were enriched in the mTOR pathway, gap junction, Jak-STAT signaling pathway, and so on. The mTOR pathway is a classic autophagy signaling pathway that plays an important regulatory role in autophagy occurrence and development. Autophagy is a catabolic process whereby cells remove damaged and dysfunctional components for nutrient recycling to deal with cellular stress and nutrient shortages. Autophagy activation could increase osteogenic differentiation and restore bone volume (Li et al., 2019). Recent work has focused on mTOR signaling as a critical mechanism responsible for chondrogenesis and osteogenesis (Wu et al., 2019). Autophagy requires the concerted activation of several Atg (autophagy-related) proteins. Autophagy-related gene 1 (ATG1 or ULK1) is critical for of autophagy initiation, and overexpression of ULK1 can activate autophagy and then increase osteoblast differentiation activity and regenerate the bone volume; subsequent activation of ULK1-dependent autophagy can be positively connected with the osteogenic differentiation of MSCs (Zhou et al., 2018; Ruolan et al., 2020; Lahiri et al., 2021).In this study, RNA-seq revealed that the mTOR signaling pathway was significantly enriched and the expressions of related genes were upregulated in the 10% CGF group, suggesting a potential regulatory role where CGF promotes osteogenic differentiation of the directly co-cultured hBMSCs by activating autophagy via the mTOR/ULK1 pathway.

In conclusion, 10% CGF exerted a significant osteogenic effect on hBMSCs directly co-cultured with HUVECs, autophagy activated by the mTOR/ULK1 pathway and CX43-mediated intercellular communication may play regulatory roles in promoting hBMSCs osteogenic differentiation.

## Data Availability

The datasets presented in this study can be found in online repositories. The names of the repository/repositories and accession number(s) can be found below: National Center for Biotechnology Information (NCBI) BioProject database under accession number PRJNA801101.
